# On Our True and False "Knowledge" of the Physiological Character and Action of the Red-Blood Corpuscles

**Published:** 1868-02

**Authors:** Rufus King Browne


### ARTICLE III.

### *On our True and False "Knowledge" of the Physiological Character and Action of the Red-Blood Corpuscles.*

(Concluded.)

By Rufus King Browne, M.D.

But the experimental demonstration of the fact, that the red-globules of the blood alone, become the appropriating agents of the oxygen of the air, in the lungs, is also demonstrative of the exact seat in the blood of the carbonic acid gas. It does this, as I think, most conclusively, by showing, that as the blood-globules which have passed the pulmonary circuit are replete with oxygen, they are *ex*cluded from being supposed to hold any of the carbonic acid contained in arterial as well as venous blood. Both bloods are well known to contain carbonic acid. On the old theory, which these various experiments contradict, that the blood-globules delivered oxygen to the tissues, suggesting that the globules received from them in interchange carbonic acid, it was but natural to suppose that they so received and carried the carbonic acid back to the lungs, for interchange with oxygen there, as they before had to the tissues, for interchange with carbonic acid. But it seems certain, that the seat in the blood of this gas must be the same in venous as in arterial blood. This seat in the latter we have shown is exclusively the plasma, by proving that in arterial blood the globules are replete with oxygen, and hence carry no carbonic acid gas.

The act of demonstrating that the globules *alone* on their way to the arterial system, take up and become the carrying seat of the oxygen, proves that the plasma is the seat of the carbonic acid in arterial blood. The demonstration that it is to the *plasma* they deliver it during their transit, and not to the tissues—dispensing entirely with that false theory,—leaves not the slightest reason for supposing that the globules take up the carbonic acid from of the plasma in which it was held. If the blood-globules receive at the pulmonary surface, the oxygen and give it to the plasma, as it advances, it is certain that the latter gives it to the tissues, not alone, but incorporated with its other substances. The carbonic acid, is derived from the tissues by the plasma which bathing them, collects in the lymph canals, to flow into the venous blood.

To still hold to the supposition that not the plasma but the red-globules, in the venous system, hold the carbonic acid, demands the supposition that the globules take it up from the plasma of venous or else arterial blood, and moreover that they discharge it into the air of the lungs, and simultaneously appropriate the oxygen. How is it conceivable that the globules can, even while the carbonic acid is issuing from them, to the pulmonary surface, also be in the act of drawing in and appropriating oxygen beyond that surface. But *all* its carbonic acid, of the blood advancing from the pulmonary tissue, is found in its plasma, and identically the same proportion of carbonic acid exists in the plasma no-arterial as it did in the plasma in the capillaries of the lungs, *if* the *globules* specially exhaled all the carbonic acid gas, given to the air in the lungs. All these circumstances are identified with the supposition that the globules, (and they are prohibited by the facts,) hold the carbonic acid during any part of the circuit.

If the blood globules do *not*, and the plasma *does* receive the carbonic acid, from the tissues, not directly but by the lymphatics, we are entirely exempt from any reason for supposing that the red-blood globules ever take it up. *They certainly do not take it up from the tissues.* While the plasma *certainly* does contain it in all the blood which passes through the lungs, and contains it where it is demonstrated the globules do not, namely: after passing the lungs and throughout the arterial system. And in no other way as I conceive, can it be more conclusively established, that the carbonic acid does not enter the globules.

Our experiments prove that it is not upon the presence of carbonic acid gas in blood, that the purple line supervenes, since instead of the presence of carbonic acid here, there must have been a loss of it ; and yet not with it, as we suppose, but *without* it, the blood lost its florid hue, on being reduced or on losing its oxygen, proving that the loss of the oxygen constitutes the loss of the scarlet. It is no less plain, therefore, that if the florid hue be certainly due to oxygen, to the loss of it, is as certainly due its reduction of color to purple, which is all simply the recurrence of the hue of its organic form and substance.

One fact is certain, that is, that the change of *color* in the red-globules of the blood, is entirely due not to its alternately taking up oxygen and carbonic acid, but to changes in the amount of oxygen alone it takes up ; this is demonstrated, and it leaves no room for the fancy, that at one moment the red-globules are completely occupied with "consuming" the oxygen, and the next moment are filled with carbonic acid. And this fancy is seen to be a mere figment of total unreality when it is observed that the most singular *in*distinction or indiscrimination and contradiction exists in the other concurring fancies of which it is an offshoot. One paragraph descriptive of these fancies makes the red-corpuscle the seat of oxydation, which gives off carbonic acid as the product of the union of its substance—its carbon and the oxygen it consumes, in these terms: "the fact that the blood corpuscles are capable *consuming* oxygen, and *giving of* carbonic acid, is an additional argument in favor of the *union* of these anatomical elements with the gas." [oxygen.] (Flint.) What kind of a *union* it is, which consists in the act of taking up oxygen and giving off carbonic acid, which arises from the union of the oxygen and the carbon of the globules, it is impossible to conceive. The union if any, here supposed, is that of the carbon and oxygen done in forming the acid gas—but this is done by the *dis*uniting of the carbon from the globule,—the essential first step of its union with the oxygen, and the "giving off" of both. It is the substances *uniting* therefore which are the seat of the union, and not the globule which gives it off.

But this only shows by its absurdity, that the blood globule cannot normally both perform the transaction of consuming oxygen and giving off carbonic acid. Although the giving off would not be an act of the oxygen, seeing that its play is over for the time being when it has united with the carbon.

But we observe also, a fancy the converse of this ; as follows :—"the plasma will absorb a *certain quantity* of oxygen ; it first takes oxygen from the air and then gives it to the corpuscles."

This fancy, though it does not coincide with the previous one, is expressly formed to coincide with the notion that in the red-globules, is produced the carbonic acid. The latter notion we have shown is fallacious. Moreover our demonstration proves that the red-corpuscles in blood, after leaving the pulmonary capillaries, yield oxygen to the plasma : and it is certainly not probable that the plasma first receives it, from the air in the lungs, to give it to the globules, again instantly to receive it back from the globules. The one statement definitely implies, that of the whole of the oxygen inhaled—the plasma absorbing only a certain quantity, the globules will absorb the rest ; and the other statement implies, that the former quantity the plasma first takes from the air, then gives it to the corpuscles. This phraseology, therefore, maintains that some of the corpuscles as well as the plasma simultaneously themselves, take oxygen from the air, while others are at the same time recipients of oxygen from the plasma.

But in observations, we ascertain that the corpuscles constitute almost the whole of the respiratory surface of the elements of the blood, in the situation where alone the oxygen is taken up, namely, in the pulmonary capillaries. The conditions do not permit our believing that *here* at least the plasma takes up the oxygen—much less that while some of the corpuscles take some, others in the same respiratory act take none, and moreover that the plasma takes up some oxygen merely for the purpose of handing it over to some of the globules.

But amid the contradictions of the same compiler we have quoted from, we find one statement, which completely *disproves*, the preceding notions altogether. "Carbonic oxide which has a great affinity for the corpuscles, displaces almost immediately, ALL the oxygen *which the blood contains.*" [*Flint. Hum. Phy.*]

From these endless contradictions and embarrassments, the only possible mode of exemption is by the demonstrations we have already adduced. Our present predicament is due to the fact, that in the absence of knowledge or even thought, we had "faith" in those mutually destructive fancies, and have formed others coincident with them.

There is one experiment I have repeatedly performed, the circumstances of which seem indubitably to establish the facts respecting the true seat of the oxygen taken from the air of the lungs. If in an animal in which the cardiac action and circulation continues after poisoning with woorara, artificial respiration be suspended, the blood in its venous state, passes through the lungs into the left ventricle. Here the failure to change in the color of the blood in passing through the lungs is indubitably due to the failure to take up oxygen. But the seat of the color and of changes of color in blood, resides wholly in its *colored constituents* the red-corpuscles. And the failure to take up oxygen during the collapsed state of the lungs, and is identical with the failure to change color from dark to bright-red. Here we do not see how, without plain and evident denial of the facts, we can evade regarding this experiment as a demonstration which disproves the notion that the plasma is ever the seat of the incoming oxygen.

But we must now recur to the second experiment we named in the beginning; the experiment, namely, that the scarlet cruorine becomes purple or venous by yielding the oxygen which makes it scarlet, to the liquid in which it is immersed whether serum or plasma. Now the substance *from* which the oxygen is taken, is not the substance which receives its supply from the fluid to which it habitually gives it. But if as, we have seen, the oxydizing process does not take place in each of the globules, at the expense and loss of so much of each of them as is supposed to be converted into carbonic acid, and if also this gas is not derived by them from the oxydation of the tissues, then the character of the process is such, (according to the supposition,) that they must deliver their oxygen into the plasma. For in order to take up carbonic acid, they must have first discharged their oxygen. This, the red-globules cannot in the nature of the case, directly impart or communicate to the tissues by bringing it into contact with their atoms, and hence they must yield it to the fluids with which they are in a contact so perfect, that the *substance* of the globule (apart from its form) is almost continuous with that of the plasma.

In the midst of these mutually contradictory and destructive suppositions, we have transcribed, we have been entirely unmindful of *our true* knowledge. This exists in a fact, long in our possession, which prohibits the entertaining of any supposition that the plasma takes up the oxygen from the air of the lungs. It definitely prohibits our acceptance of the notion, that the liquor sanguinis takes up the oxygen first from the air of the lungs and then serves it to the globule. This fact is, that, the liquor sanguinis, will not hold more than a 1-15 part of the oxygen the globules will, and hence the former *cannot* take up the proportion, to fill the globules, nor the proportion of oxygen actually taken up.

This fact definitely disposes of that eggregiously absurd supposition, that the liquor sanguinis and globules, *both* take up and carry both oxygen and carbonic acid: and taken together with the fact demonstrated by Bernard, that carbonic oxide in uniting with the redglobules, for which it has a remarkable affinity, displaces *all* the oxygen of arterial blood, proves that it does not perform the office of *displacing* carbonic acid from the liquor sanguinis.

It is not really conceivable, that the globules discharge the carbonic acid, for it implies, since we know that they take the oxygen, that they both discharge carbonic acid, and take in the oxygen in the twinkling of an eye.

And all the demands of our intelligence are completely met upon this point, in the fact that the globules alone will absorb fifteen times more oxygen than the plasma, and hence the latter cannot possible take up as they are said to, and impart to the latter, the proportion of oxygen contained in arterial blood.

The only possible escape from all this endless embarassment, is to be found in the experimental evidence named throughout this article, that the oxygen is solely taken up and carried by the red-globules, and by it delivered to the plasma. That the latter is the exclusive carrier of the carbonic acid of the blood, and that the change of color from arterial to venous blood, is entirely due to the loss of oxygen by the globules to the plasma.

We have always considered this the case of one substance absorbed in the globule producing *one* color—purple, which had to be discharged or evacuated by another substance producing another color,—scarlet. If it were, as we *must* erroneously suppose, that the purple color was induced by the entrance into the globule of some substance, as the scarlet color is by that of oxygen, then the purple color could only occur by actual displacement of oxygen in it, by carbonic acid. But this purple remains under circumstances in which the carbonic acid gas is *not present*, and will only become scarlet on access of oxygen, showing that the change of color is not due to *impletion* of carbonic acid, but to access of oxygen. The same truth is apparent in the use of hydrogen, nitrogen, and carbonic acid. The red-corpuscle of venous blood

*remains* purple, in the absence of oxygen, though there be no carbonic acid present in it. Showing that the presence of carbonic acid in it, is not the *cause* of that color.

The venous hue, then, is not another and second color produced by the presence in the globule of another and different substance, which displaced the oxygen from it, and thereby gives a new color to it, driving out the scarlet, .which changes the scarlet, to purple simply because they cause the *loss* of the oxygen.

The presence of the scarlet hue presupposes the prior *existence* of the purple hue, due simply to loss of oxygen. I have said this was not a process of "oxydation," as presented in the supposition that the globules consume oxygen, and "*give off*" carbonic acid, and this is plain to the very least intelligence, and the acceptance of a *contrary* supposition, shows how little intelligence we have exercised. By this supposition it is presupposed that the red globules first take up the carbonic acid from the rest of the blood or the tissues, and give it to the plasma (which is contradicted by another supposition, that the carbonic acid is formed by oxydizing of their own substance,) or else that the carbonic acid they give off, is that *portion* of themselves which had become carbonic acid by oxydation. But the first of these is contradicted by another supposition, viz.: that it is the plasma, not the globules, from which the oxygen of the air displaces the carbonic acid in the lungs. And the second itself is contradicted by the fact, which satisfies all the demand of our knowledge, that the anatomical form of the globules could not be maintained, during a single round of the circulation, if they really were oxydized, or their substance converted into *carbonic acid*, or any product of oxydation. In which case they could not take up oxygen a *second* time, which presupposes in the meantime, that the globules are devoid both of carbonic acid, that being in the plasma, when it is displaced and not in them ; and devoid of oxygen, until the regular course of proceeding of displacing the carbonic acid, taking up the oxygen, and giving it up to them. At every new sentence we are confronted by some one of these contradictory statements, and no one of them is uncontradicted by some other one.

An example, that we constantly make statements which we have not exercised the least intelligence of the character of, is found in the statement, that "the elements of the blood which absorb the *greater part* of the oxygen, are the red-corpuscles."—(*Flint. Hum. Phy.*)

As if some of these corpuscles coming nearest to the air did, and others did *not*, (or were prevented from), absorbing oxygen. And the error is worse when we consider that the plasma could not in fact give to these globules a fraction of one per cent. of the oxygen they themselves are found to hold. How too are the globules going to get it? Here are a set of bodies, most peculiarly fitted for this work of taking up oxygen, without which we should be absolutely at a loss, to account for its being taking up in respiration. They will and *do* take up fifteen times as much as the liquid in which they are suspended. Their form and dimensions,—all their anatomical characters, are precisely those which show peculiar adaptation to this function. And, yet while we are in the act of regarding *these facts*, we say that they take up the oxygen which is supplied by asserting that they only take up the "greater part" of the oxygen, and the plasma the rest. Look at the enormous surface they present to the approach of the oxygen as compared with a fluid surface, say of the linear measure of a fraction of one inch. And if the oxygen come in contact with various points of the surface of each of them the corresponding number of atoms of the oxygen, over that of the plaen surface of a fluid, is enormously increased. Still, we suppose the globules do not take up the oxygen *actually* taken up, but only the "greater part" of it, and even this, itself the statement of an error, we contradict by saying that "the plasma first takes up the oxygen and gives it to the globules."

According to this most egregious of all blunders, we have to suppose either, that only a certain proportion or number of globules present in the lung capillaries, during each inspiration, take up the due proportion of oxygen they are found to have, while some other of the globules, do not take any, except as furnished afterward by the plasma, or else that the globules do not directly of themselves take up their due amount of oxygen, but taking up directly a certain deficient amount, the plasma gives up to them what constitutes the complement of the due proportion they hold.

But this supposition is contradicted by another, the absurdity of which is, if possible, still more monstrous, namely, that the plasma first takes the oxygen and then gives it to the globules. (*Flint. Hum. Phy.*) Now, this is said of the plasma, which, according to the same author, who adopts both the preceding suppositions,—will take up about 1-15th the amount of oxygen the globules will. So that, if the plasma, first takes the oxygen during inspiration, it must absorb as much as it can, *fifteen times in succession, each act of inspiration*, in order to supply the globules with the amount they will hold. Since, however, the plasma cannot in inspiration take up more than once, the small percentage it holds, this supposition requires us to believe that, if the plasma, first takes up the oxygen from the air, that the sole amount of oxygen the globules get is about six per cent. of what *in fact* they actually contain after passing through the pulmonary capillaries ; and the same want of intelligence, is shown in an interpretation put upon an experiment of Stevens, who, "After removing the serum from a clot by repeated washings, with pure water, found that the color remained black when exposed to the air, but was reddened by the addition of its serum From this he reasoned that the red color of the arterial blood, is due to the saline constituents of the plasma."

Comment: "This is true, but the saline constituents of the plasma, affect the color indirectly, by *maintaining* the anatomical integrity of the corpuscles.—(*Flint. Hum.*

*Phy. Cir. p.* 456.) The *facts* of the experiment are not even hinted at or implicated in the interpretation of the author who cites it. For the plasma was not permitted to hold the globules, and was, therefore, especially removed from any effect of "*maintaining*" the anatomical integrity of the corpuscles. The globules were specially *deprived* of this condition of their integrity, and no means was employed to preserve it, nor could the serum used have possibly *preserved* this integrity, for it was not used until the change was actually accomplished. Whatever the effect, which occurs, of the separation of the globules and plasma, actually *did* occur in this instance, and yet the interpretation put upon it, ignores all the facts.

And will then, dark *venous* blood *redden* merely by keeping up its "anatomical integrity," or worse than this, will this blood when it has *not* changed from venous hue to red, on exposure to both air and oxygen, in time *redden* on contact with serum, without any other cause than this, *erroneously* supposed, preservation of the integrity of the corpuscles, which we have shown was not the characteristic fact of the experiment? The answer to this is plain. The change of color by the serum, was wholly due to the oxygen it contained, derived from the clot itself. But however this may be, the interpretation put upon the experiment by the author we have quoted, exemplifies an entire want of intelligence in regarding the facts. Such monstrous absurdities as these, we say, credited in the very presence of the facts, show that our intelligence *has not yet come to the birth* regarding them; for it is plain to even an instant exercise of intelligence, that no wilder errors could be imagined.

In any situation, in or out of the blood channels, the red blood corpuscle shows palpably to the senses, the possession of truly organized characters. The changes of contour and character so far as these are appreciable to sense it invariably undergoes, even where it is carefully and effectually protected from gross physical influences are not due to any *action* upon it, of outward agents by which it is deprived of its wet constituents, but are the variations in an organic form prior to final passage from organic to exorganic substance bnt which is one of inert waste. These changes show how widely different its normal state is from any mere inert mass of matter. These changes would probably be prevented rather than be *caused* by such external influences. As they exist, however, out of the body, and it goes through certain characteristic stages of changes, even if we consider the last it comes to as one of inertia or organic death—it is demonstrated that it commenced these changes in a very different state, from that of inertia. From one state it undergoes a series of abrupt changes which ultimate in another state. If the latter is one of inertia or organic decease, the former was *not*, for the changes mark the passage from life to death. Surely these changes take place, *not* in inert matter only chemically formed and moved, but in the most highly organized matters;—i. e. in substances, which not alone in material constitution, but in *character*, are vivid. The failure to recognize this truth is, I am convinced, explicable on no other assumption, than that we habitually, whether from a state of childish ignorance or not—*fail to reflect for a moment* on facts of this character. The fact of the changes which demonstrate the great difference between the red-blood corpuscle and any kind of matter of chemical nature and *not* vivid, are precisely the same, whether we attribute them to outer influence or not. That they would not so change in the blood channels only furnishes another kind of proof or demonstration of their highly vivid character, for there they maintain it for a certain definite term of life.

---

